# Supplementary material for: A Qualitative Study on the Barriers and Enablers to Effective Hypertension Management in Ghana
Source: Healthcare (Basel). 2025 Feb 22;13(5):479. doi: 10.3390/healthcare13050479 (PMC11898504; doi:10.3390/healthcare13050479)
Supplement: Supplementary file 1 [file healthcare-13-00479-s001.zip › healthcare-3434593-supplementary.pdf]

Supplementary File

POTENTIAL INTERVIEW QUESTIONS FOR HYPERTENSION PATIENTS

SECTION A: MEDICATION ADHERENCE

1. Tell me your experience when you were first diagnosed with hypertension (HPT). Probe (acceptance of the condition, living with the condition for life)
2. How will you describe your hypertension management? Probes (medication adherence, lifestyle modification, social support).
3. What are the barriers you face in taking your antihypertensive medications? Probe (barriers to adherence, e.g., side effects, shortage of medication, forgetfulness, lack of social support from family members to prompt on taking the drugs, money to purchase hypertension medication, barriers from government, barriers from healthcare providers).

   Probe (ability to bring blood pressure under control, uncontrol blood pressure).
4. Tell me about the opportunities that can help enhance your ability to manage your hypertension condition effectively.
5. Tell me how you have been taking your medication Probe (times a day per the physician prescription, always/adherence, non-adherence).

   Probe (non-prescribe medications e.g., pain killers, herbs, spiritual water from religious affiliations)

SECTION B: KNOWLEDGE OF HPT CONTROL

1. Tell me what you know about hypertension Probe (blood pressure threshold)
2. What do you think is the source of your hypertensive illness? Probe (false beliefs, myths, misconceptions, risk factors)
3. How are you treating your hypertension? Probe (use of hospital prescribed antihypertensive medication, self-medication, not taking medication)

SECTION C: LIFESTYLE MODIFICATION

1. Tell me about some of the lifestyle behaviours you are expected to change due to your hypertensive condition?

Probe (Alcohol, physical inactivity, salt intake, stressful situations, sleep)

2. How difficult is it for you to change these lifestyle behaviours that affect your hypertension control?
3. What can you do better to enhance your efficacy over these lifestyle challenges?

4. To what extent do you feel supported by your government in the management of your condition?
5. To what extent do you feel supported by the healthcare providers in the management of your HPT condition?

Is there anything you may want to share with me?

Thank you for your attention and cooperation
